# Loss of the Wnt receptor frizzled 7 in the mouse gastric epithelium is deleterious and triggers rapid repopulation *in vivo*

**DOI:** 10.1242/dmm.029876

**Published:** 2017-08-01

**Authors:** Dustin J. Flanagan, Nick Barker, Cameron Nowell, Hans Clevers, Matthias Ernst, Toby J. Phesse, Elizabeth Vincan

**Affiliations:** 1 University of Melbourne and Victorian Infectious Diseases Reference Laboratory, Doherty Institute of Infection and Immunity, Melbourne, Victoria 3000, Australia; 2 Institute of Medical Biology, Singapore 138648, Singapore; 3 MRC Centre for Regenerative Medicine, University of Edinburgh, Edinburgh EH8 9YL, UK; 4 Monash Institute of Pharmaceutical Sciences, Parkville, Victoria 3052, Australia; 5 Hubrecht Institute for Developmental Biology and Stem Cell Research, 3584CT Utrecht, Netherlands; 6 Olivia Newton-John Cancer Research Institute, Australia and La Trobe University School of Cancer Medicine, Heidelberg, Victoria 3084, Australia; 7 European Cancer Stem Cell Research Institute, Cardiff University, Cardiff CF24 4HQ, UK; 8 School of Biomedical Sciences, Curtin University, Perth, WA 6845, Australia

**Keywords:** Frizzled7, Fzd7, Gastric homeostasis, Wnt

## Abstract

The gastric epithelium consists of tubular glandular units, each containing several differentiated cell types, and populations of stem cells, which enable the stomach to secrete the acid, mucus and various digestive enzymes required for its function. Very little is known about which cell signalling pathways are required for homeostasis of the gastric epithelium. Many diseases, such as cancer, arise as a result of deregulation of signalling pathways that regulate homeostasis of the diseased organ. Therefore, it is important to understand the biology of how normal conditions are maintained in a tissue to help inform the mechanisms driving disease in that same tissue, and to identify potential points of therapeutic intervention. Wnt signalling regulates several cell functions, including proliferation, differentiation and migration, and plays a crucial role during homeostasis of several tissues, including the intestinal epithelium. Wnt3a is required in the culture medium of gastric organoids, suggesting it is also important for the homeostasis of the gastric epithelium, but this has not been investigated *in vivo*. Here, we show that the Wnt receptor frizzled 7 (Fzd7), which is required for the homeostasis of the intestine, is expressed in the gastric epithelium and is required for gastric organoid growth. Gastric-specific loss of *Fzd7* in the adult gastric epithelium of mice is deleterious and triggers rapid epithelial repopulation, which we believe is the first observation of this novel function for this tissue. Taken together, these data provide functional evidence of a crucial role for Wnt signalling, via the Fzd7 receptor, during homeostasis of the gastric epithelium.

## Abstract (precis)

**Editors’ choice:** Wnt signalling regulates homeostasis of the gastric epithelium via the Fzd7 receptor, which could be a target for therapeutic intervention in gastric cancer.

## INTRODUCTION

The Wnt signalling pathway regulates multiple cellular functions, including proliferation, migration, differentiation and stem cell function (Clevers and Nusse, 2012), and is crucial during embryonic development (van Amerongen and Nusse, 2009). The control of important cellular functions by Wnt signalling is maintained into adulthood, during which several organs require Wnt signalling for correct homeostasis, including the intestinal tract, hair follicle, mammary gland and liver (Clevers et al., 2014). Wnt signalling has to be tightly regulated during homeostasis, as deregulated Wnt signalling is often one of the earliest oncogenic events in several cancers (Polakis, 2012).

Wnt signalling is divided into three broad pathways: canonical β-catenin, planar cell polarity (PCP) and calcium-dependent signalling (Wnt/Ca^2+^) (Niehrs, 2012). The cytoplasmic signal transducers that regulate these pathways have been the subject of intense research interest, and in the case of the canonical β-catenin pathway, its role is well characterised in several biological contexts, including development, homeostasis, regeneration and cancer, particularly in the intestine.

The receptors that transmit Wnt signalling are beginning to be understood in more detail, with a complex model emerging in which frizzled (Fzd) Wnt receptors associate with various co-receptors to activate different Wnt pathways (Niehrs, 2012). To date, 19 Wnt ligands and 10 Fzd receptors have been discovered in mammals, and the Wnt pathway is highly conserved from humans through to evolutionarily older organisms, including *Hydra* (Nichols et al., 2006; Srivastava et al., 2008).

The gastric epithelium is composed of parallel, glandular invaginations known as gastric units. Each gastric unit is composed of a pit, which is continuous with the surface epithelium and a flask-shaped gland, which extends down further into the isthmus, neck and base areas. Distinct areas within individual gastric units are characterised by the residency of specialised cell types that regulate various aspects of digestion: gastric mucus cells that secrete protective mucus; parietal cells responsible for secreting hydrochloric acid; chief cells that release active pepsin; and several types of endocrine cells that secrete an array of hormones that aid and regulate digestion and absorption, including ghrelin and somatostatin (Mills and Shivdasani, 2011). Importantly, the precise architecture, cellular heterogeneity and turnover rate of the gastric units varies markedly between the two major anatomical regions of the stomach, the antrum and corpus (Mills and Shivdasani, 2011).

Several studies have implicated Wnt signalling as being important in the gastric epithelium, although its role is poorly understood in comparison with that in the intestinal epithelium. Gastric organoid cultures require Wnt3a in the culture medium in addition to the Wnt agonist R-spondin (Barker et al., 2010; Flanagan et al., 2016), demonstrating that Wnt is required for the gastric epithelium. The R-spondin receptor Lgr5 is expressed in cells that respond to Wnt signals and is a marker of stem cells in several organs, including the gastric epithelium (Barker et al., 2010; de Lau et al., 2011), demonstrating that Wnt-responsive stem cells reside in the gastric epithelium. The Wnt pathway is more active in the antrum than in the corpus; however, *Troy*^+^ (*Tnfrsf19*^+^) cells in the corpus express Wnt target genes and stem cell signature genes (Stange et al., 2013). Experimental deregulation of Wnt signalling in the gastric epithelium can also result in tumourigenesis (Radulescu et al., 2013), similar to the intestinal epithelium (Sansom et al., 2004).

We have recently demonstrated that Fzd7 is the predominant Wnt receptor in regulating homeostasis in the intestinal epithelium, in which deletion of *Fzd7* in either the whole epithelium or specifically in the *Lgr5*^+^ intestinal stem cells, triggers rapid repopulation (Flanagan et al., 2015). Here, we show that *Fzd7* is also expressed in the antrum of the gastric epithelium, and is required for the growth of gastric organoid cultures. Deletion of *Fzd7* in the gastric epithelium *in vivo* was deleterious and triggered rapid repopulation of the epithelium – the first time repopulation has been reported for the stomach following a genetic insult. These data identify that Fzd7 is crucial for transmitting Wnt signalling to regulate homeostasis in the gastric epithelium.

## RESULTS

### Wnt signalling is required for gastric homeostasis

**Fig. 1.**
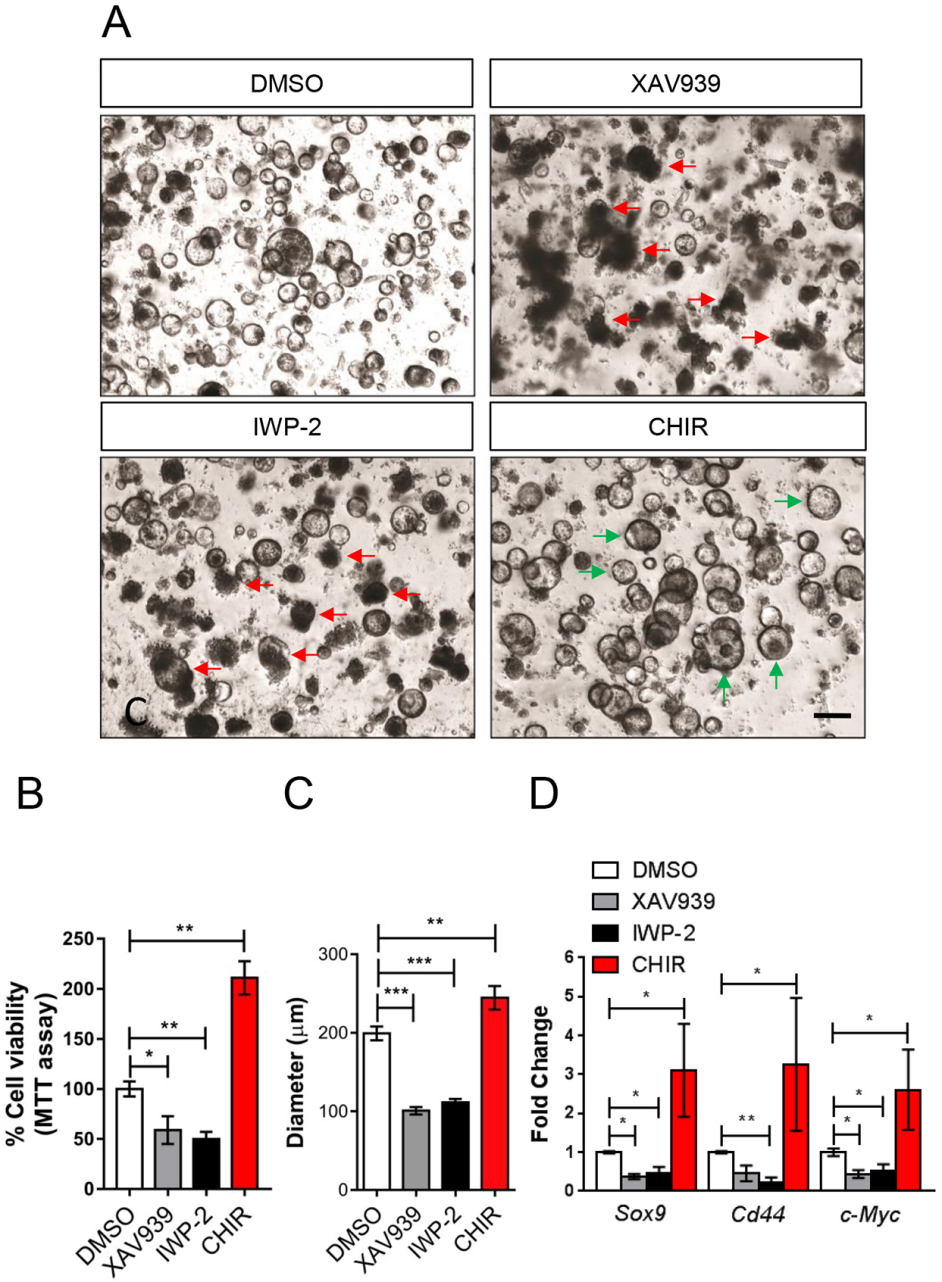
**Wnt signalling is required for gastric epithelial cell growth.** (A) Organoids cultured from antral epithelium of wild-type mice treated with vehicle (DMSO), tankyrase inhibitor (XAV939), porcupine inhibitor (IWP-2) or Gsk3β inhibitor (CHIR-99021). Organoids were cultured for 3 days and treated for 48 h before pictures were taken. Green arrows indicate live organoids; red arrows identify dead/dying organoids. Scale bar: 200 µm. (B) MTT assay for cell viability of the organoids described in A. Three mice were used per experimental condition; each experiment was performed separately three times using six replicates of each condition (*P<0.05, **P<0.01; data are mean±s.e.m., *n*=3 mice, Mann–Whitney). (C) Measurement of wild-type gastric organoids following treatment with compounds as described in A. Measurements were performed using ImageJ analysis software (***P<0.001, **P<0.01; data are mean±s.e.m., *n*=3 mice, minimum of 100 organoids measured per mouse, Mann–Whitney). (D) qRT-PCR for Wnt/β-catenin target genes from organoids described in A (*P<0.05, **P<0.01; data are mean±s.e.m., *n*=3 mice, Mann–Whitney).

Wnt signalling is crucial for homeostasis of the small intestine (Clevers and Nusse, 2012; Flanagan et al., 2015); however, it is less well understood in the gastric epithelium. To examine the requirement for Wnt signalling in the gastric epithelium, we established organoid cultures from the mouse antral gastric epithelium and exposed them to various Wnt pathway inhibitors and activators, which we validated via TOPFLASH assays (Molenaar et al., 1996) and western blots for active β-catenin (van Noort et al., 2002) in HEK293 cells (Fig. S1A,B). Organoids treated with either the porcupine inhibitor IWP-2, which prevents secretion of Wnt ligands (Chen et al., 2009), or the tankyrase inhibitor XAV939, which stabilises the β-catenin degradation complex and consequently inhibits Wnt signalling (Huang et al., 2009), underwent rapid atrophy and organoid death. This was not observed in vehicle-treated organoids, which continued to thrive (Fig. 1A). Conversely, gastric organoids treated with the selective Gsk3β inhibitor CHIR-99021 (CHIR), thereby activating Wnt signalling (Bennett et al., 2002), showed increased organoid size and viability (Fig. 1A-C). These observations were supported by a thiazolyl blue tetrazolium bromide (MTT) assay showing marked reduction in cell viability in gastric organoids treated with either XAV939 or IWP-2, and conversely increased metabolism in organoids treated with Wnt agonist CHIR (Fig. 1B). Quantitative reverse transcriptase PCR (qRT-PCR) was then performed on total RNA extracted from the treated gastric organoids, identifying that expression of Wnt target genes *Sox9*, *Cd44* and *Myc* was significantly reduced following XAV939 or IWP-2 treatment, and was conversely upregulated following CHIR treatment (Fig. 1D). Interestingly, expression of Fzd genes is increased in organoids treated with IWP-2 or XAZ939, presumably as a mechanism to increase Wnt signalling in response to these compounds inhibiting the pathway, but as Wnt target genes are still reduced, this response is insufficient to activate Wnt signalling and thus the organoids die (Fig. S1C). Collectively, these data demonstrate that Wnt signalling is crucial for gastric organoid growth and maintenance, and they identify that Wnt ligands secreted from the epithelial cells of the gastric organoids are required cell-autonomously for their growth and survival. This strongly implicates an integral role for Fzd receptors in transmitting these essential Wnt signals in the gastric epithelial cells.

### Fzd7 is expressed in the gastric epithelium

**Fig. 2.**
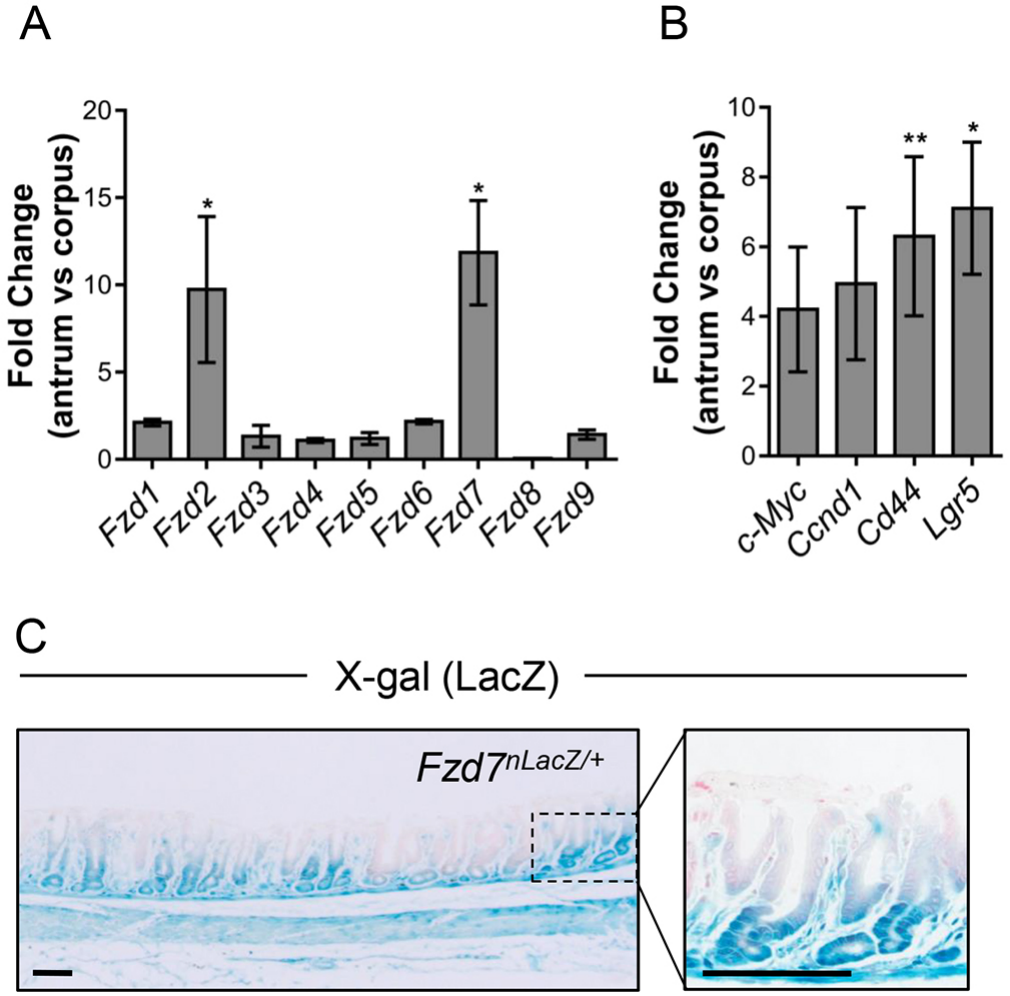
***Fzd7* is expressed in the gastric epithelium.** (A) qRT-PCR of *Fzd* receptors in the antrum and corpus epithelium of the adult mouse stomach (*P<0.05; data are mean±s.e.m., *n*=4 mice, Mann–Whitney). (B) qRT-PCR of Wnt/β-catenin target genes indicated in the antrum and corpus epithelium of the adult mouse stomach (*P<0.05, **P<0.01; data are mean±s.e.m., *n*=4 mice, Mann–Whitney). (C) X-gal staining of the antral stomach of *Fzd7^nLacz/+^* mice. Dotted box outlines the region magnified on the right. Scale bars: 50 µm.

Little is known regarding the extent of Wnt signalling and the expression of Fzd receptors in the gastric epithelium. To investigate the expression of Fzd receptors in the gastric epithelium, we extracted RNA from the antrum and corpus epithelium of wild-type mice and performed qRT-PCR. The expression of *Fzd2* and *Fzd7* was markedly higher in the antrum than in the corpus, both comparatively (Fig. 2A) and in the raw data (Fig. S2A). Fzd7 is of particular interest as it is required for embryonic stem cell activity (Melchior et al., 2008) and we have recently shown it to be the predominant Wnt receptor regulating homeostasis in the intestinal epithelium (Flanagan et al., 2015). The increase in *Fzd7* expression, a Wnt target gene (Vincan et al., 2010), in the antrum is also associated with an increase in the expression of other Wnt target genes, including *Myc*, cyclin D1 (*Ccnd1*), *Cd44* and *Lgr5* (Fig. 2B and Fig. S2B), suggesting Fzd7 is transmitting Wnt signalling in the isthmus and base of the antral glands. Although the expression of *Lgr5* is approximately sevenfold higher in the antrum compared with the corpus, its comparative expression with other Wnt targets in the antrum is relatively much lower, reflecting its function as a stem cell marker (Fig. S2B). To visualise the expression of *Fzd7* in the gastric epithelium, we performed X-gal stains on stomachs isolated from *Fzd7^nLacZ/+^* mice, which express the *LacZ* gene under the control of the endogenous *Fzd7* regulatory region (Yu et al., 2012). Staining was observed from the base of the antral gastric glands to the isthmus, illustrating that *Fzd7* is expressed in these cells (Fig. 2C).

### Fzd7 is required for the culture of gastric organoids

A powerful tool to understand gene function in a particular tissue is to be able to conditionally delete it specifically in the tissue of interest. To drive genetic recombination in the gastric epithelium, we used the tamoxifen-inducible *Tff1Cre^ERT2^* mouse (Thiem et al., 2016). These mice provide robust recombination in the antral glands, with recombined glands still observed 30 days after tamoxifen induction, demonstrating that recombination must occur in a stem cell population (Fig. S3). As previously reported, some cells are also recombined in the pit region of the corpus but these cells do not give rise to entire glands, suggesting that recombination does not occur in a stem cell with the capacity to populate this tissue with all the differentiated lineages observed (Thiem et al., 2016) (Fig. S3). However, a few recombined cells are still observed long after the continuous renewal of the corpus has replenished the gland cell population, suggesting either a long-lived population of non-stem cells is recombined here or, alternatively, recombination occurs in a population of stem cells here that only gives rise to a small restricted population of cells in the corpus.

**Fig. 3.**
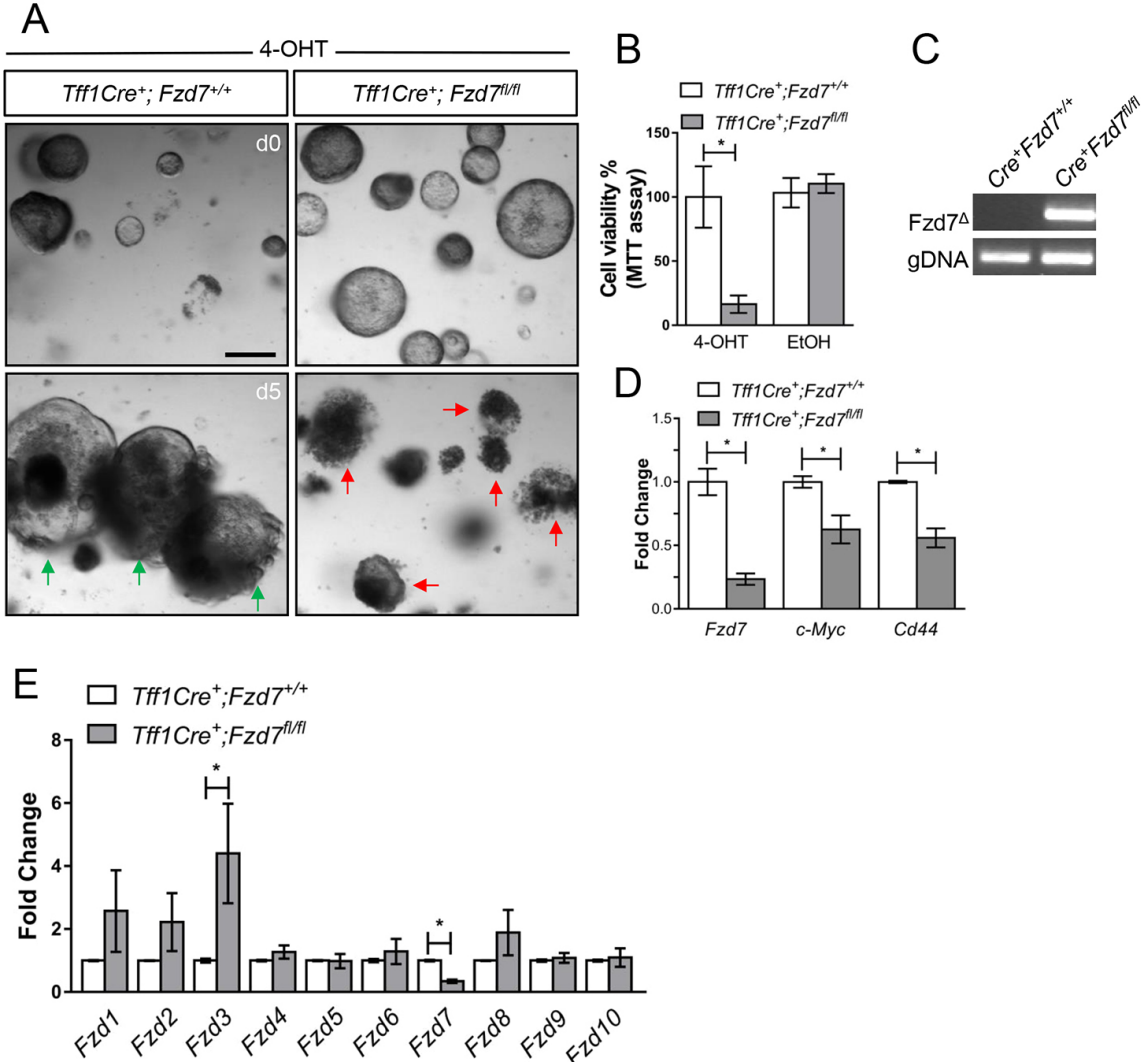
***Fzd7* is required for gastric organoid survival and maintenance.** (A) Organoids grown from indicated genotypes treated with 4-OHT (tamoxifen) at day 0 (d0) and day 5 (d5) after treatment. Green arrows indicate viable organoids; red arrows identify dead/dying organoids. Scale bars: 200 µm. (B) MTT assay for cell viability of the organoids described in A. Three mice were used per experimental condition; each experiment was performed separately three times using six replicates of each condition (*P<0.05, data are mean±s.e.m., *n*=3 mice, Mann–Whitney). (C) Conventional PCR to detect the deleted product of Fzd7 (Fzd7^Δ^) in organoids from the genotype indicated 3 days after treatment with 4-OHT. (D) qRT-PCR for Wnt/β-catenin target genes from organoids described in A 5 days after 4-OHT treatment (*P<0.05; data are mean±s.e.m., *n*=3 mice, Mann–Whitney). (E) qRT-PCR for Fzd receptors on organoids described in A, 3 days after treatment with 4-0HT (*P<0.05; data are mean±s.e.m., *n*=3 mice, Mann–Whitney).

To investigate the requirement for *Fzd7* in the gastric epithelium, we grew gastric organoid cultures from the antrum of *Tff1Cre^ERT2/+^; Fzd7^flox/flox^* mice (*Tff1Cre^+^; Fzd7^fl/fl^*) to enable tamoxifen-induced deletion of *Fzd7*. Five days after administration of 4-OHT (the metabolically processed version of tamoxifen) to the medium, we observed widespread atrophy of gastric organoids derived from *Tff1Cre^+^; Fzd7^fl/fl^* mice, whereas gastric organoids derived from *Tff1Cre^+^; Fzd7^+/+^* mice continued to thrive (Fig. 3A). These observations were supported by a MTT assay, which showed a significant reduction in organoid viability following *Fzd7* deletion (Fig. 3B) and no changes in gastric organoids treated with vehicle only (Fig. S4). To confirm robust deletion of *Fzd7* we performed PCR on genomic DNA (gDNA) isolated from organoids 3 days after 4-OHT, when organoids were still alive (Fig. 3C). The recombined *Fzd7* allele (Fzd7^Δ^) showed a very strong amplified product in the organoids derived from *Tff1Cre^+^; Fzd7^fl/fl^* mice, and was undetectable in organoids derived from *Tff1Cre^+^; Fzd7^+/+^* mice (Fig. 3C). These data demonstrate robust deletion of *Fzd7*, which was confirmed by performing qRT-PCR at the same 3-day time point, showing ∼80% reduction of *Fzd7* and downregulation of Wnt target genes *Myc* and *Cd44* (Fig. 3D). Previously, we have observed upregulation of *Fzd1* and *Fzd2* expression to partially compensate for the experimental deletion of *Fzd7* during intestinal regeneration (Flanagan et al., 2015). Expression analysis of *Fzd* genes revealed that *Fzd1*, *Fzd2* and *Fzd3* levels were elevated in gastric organoids following *Fzd7* deletion, although only *Fzd3* was significantly different in expression (Fig. 3E). As the organoids still undergo atrophy and die 3 days post-deletion of *Fzd7*, this suggests that these elevated Fzd receptor levels are unable to compensate for the loss of *Fzd7* in this system. Together, these data demonstrate that Wnt signalling is required for the maintenance of gastric epithelial cells *ex vivo* via the Wnt receptor Fzd7.

### Deletion of *Fzd7* in the gastric epithelium *in vivo* is deleterious and triggers repopulation

**Fig. 4.**
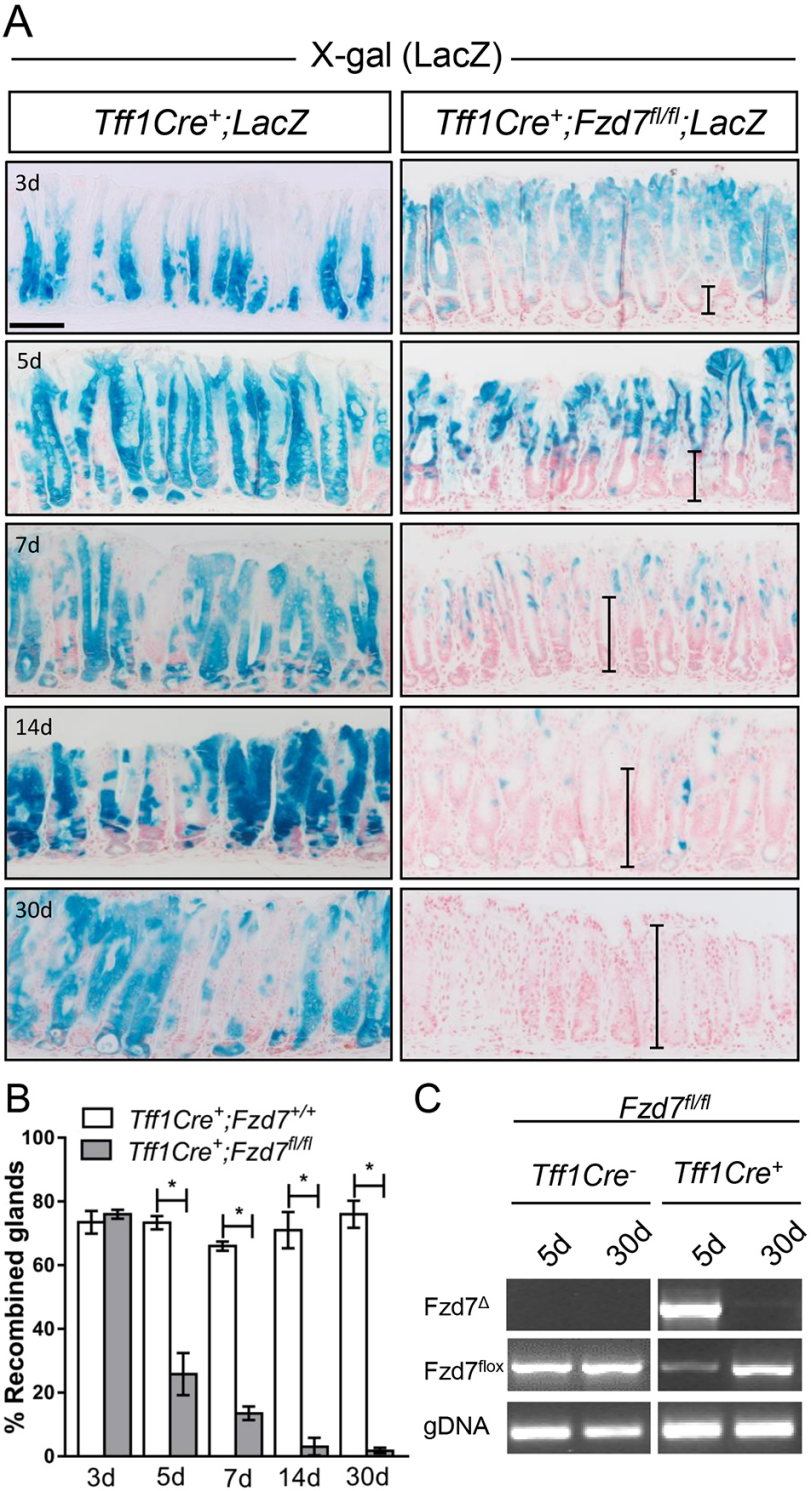
**Deletion of *Fzd7* triggers epithelial repopulation in the antral stomach.** (A) X-gal staining on antral sections of mice of genotypes indicated over the course of 1 month (d=days post-induction with tamoxifen). Vertical bars indicate the extent of repopulation. Scale bar: 200 µm. (B) Enumeration of recombined gastric glands in the genotypes indicated over time following tamoxifen injection. A minimum of 40 glands was scored per mouse (*P<0.05; data are mean±s.e.m., *n*=4 mice, Mann–Whitney). (C) PCR to detect recombined product of Fzd7 (Fzd7^Δ^) and un-recombined Fzd7flox allele (Fzd7^flox^) from genomic DNA isolated from antral epithelium from mice of the genotypes and time points indicated.

Deletion of *Fzd7* in organoids derived from the intestinal epithelium resulted in widespread crypt atrophy and organoid death (Flanagan et al., 2015). Interestingly, when *Fzd7* was deleted in the intestinal epithelium *in vivo*, it did not result in denuding of the epithelium, which might have been expected, but instead triggered repopulation of the entire epithelium with non-recombined *Fzd7*-proficient cells (Flanagan et al., 2015). Although this has been observed previously with deletion of other important genes in the intestinal epithelium, including *Myc* (Muncan et al., 2006) and *Chek1* (Greenow et al., 2009), repopulation has never been observed in the gastric epithelium. To determine the requirement for *Fzd7* in the gastric epithelium *in vivo*, we conditionally deleted *Fzd7* using *Tff1Cre^+^; Fzd7^fl/fl^*; *Rosa26LacZ^LSL^* mice in which we could track the fate of recombined cells in the stomach over time. At 3 days after tamoxifen induction *Tff1Cre^+^; Fzd7^+/+^*; *Rosa26LacZ^LSL^* mice displayed lineage tracing throughout the glands of the antrum; however, *Tff1Cre^+^; Fzd7^fl/fl^*; *Rosa26LacZ^LSL^* mice had slightly less recombination at this time point in the base of the glands (Fig. 4A). At 5 days after tamoxifen induction, *Fzd7*-deleted *Tff1Cre^+^; Fzd7^fl/fl^*; *Rosa26LacZ^LSL^* mice had markedly fewer recombined cells in the lower halves of the glands, whereas *Tff1Cre^+^; Fzd7^+/+^*; *Rosa26LacZ^LSL^* mice continued to contain recombined cells throughout the glands (Fig. 4A). The replacement of *Fzd7* recombined cells with non-recombined cells continued over time until the entire epithelium was repopulated by non-recombined (pink) cells (Fig. 4A). Enumeration of this event shows a rapid loss of recombined (blue) glands from 5 days after tamoxifen induction, leading to total repopulation of the epithelium at 30 days (Fig. 4B).

To genetically monitor the repopulation event, we isolated gDNA from the antral epithelium at 5 and 30 days post-tamoxifen induction and performed PCR for the genetic product of the deleted recombined *Fzd7* allele (*Fzd7*^Δ^) and also the non-recombined *Fzd7* flox allele (*Fzd7*^flox^). At 5 days after tamoxifen injection there was a large amplification of the *Fzd7*^Δ^ product in the *Tff1Cre^+^; Fzd7^fl/fl^* mice that was undetectable in the Cre-negative *Tff1Cre^−^; Fzd7^fl/fl^* mice at the same time point. This indicates that the *Fzd7* flox allele had undergone robust recombination only in tamoxifen-treated *Tff1Cre^+^; Fzd7^fl/fl^* mice (Fig. 4C). This was confirmed by a weak non-recombined product for the *Fzd7*^flox^ allele in the *Tff1Cre^+^; Fzd7^fl/fl^* mice [some non-recombined product is still present due to the recombination not occurring in 100% of the cells of the gastric antrum (Fig. 4B)], compared with a strong non-recombined *Fzd7*^flox^ product in the *Tff1Cre^−^; Fzd7^fl/fl^* mice 5 days after tamoxifen (Fig. 4C). At 30 days after tamoxifen, the *Fzd7*^Δ^ product is almost undetectable in *Tff1Cre^+^; Fzd7^fl/fl^* mice, which coincides with a marked increase of the non-recombined *Fzd7*^flox^ product, demonstrating the repopulation of the gastric epithelium with non-recombined *Fzd7*-proficient cells (Fig. 4C). Conversely, the non-recombined *Fzd7*^flox^ product remains strong and unchanged 5 days and 30 days after tamoxifen treatment in the *Tff1Cre^−^; Fzd7^fl/fl^* mice, indicating no recombination of this allele and thus no deletion of *Fzd7* (Fig. 4C). These data molecularly demonstrate that deletion of *Fzd7* in the antrum of the gastric epithelium is a deleterious event and triggers rapid repopulation with *Fzd7*-proficient cells.

To investigate whether repopulation could be triggered by deletion of a different Fzd receptor, we deleted *Fzd5* in the gastric epithelium (Fig. S5A). In contrast to deletion of *Fzd7*, we did not observe any repopulation after tamoxifen induction in *Tff1Cre^+^; Fzd5^fl/fl^*; *Rosa26LacZ^LSL^* mice, with recombined cells still present 30 days after deletion (Fig. S5A). Deletion of *Fzd5* was confirmed by qRT-PCR on cDNA isolated from the antral gastric epithelium, which showed a significant reduction in *Fzd5* expression, but no change in the expression of the canonical Wnt target genes *Ccnd1*, *Myc*, *Cd44* and *Lgr5* (Fig. S5B). These data demonstrate that Fzd5 is not required for gastric homeostasis and suggests that the phenotype observed when we delete *Fzd7* is not a generic event triggered by deletion of any Wnt receptor from the stomach.

### Fzd7 regulates differentiation and cell position in the gastric antral epithelium

**Fig. 5.**
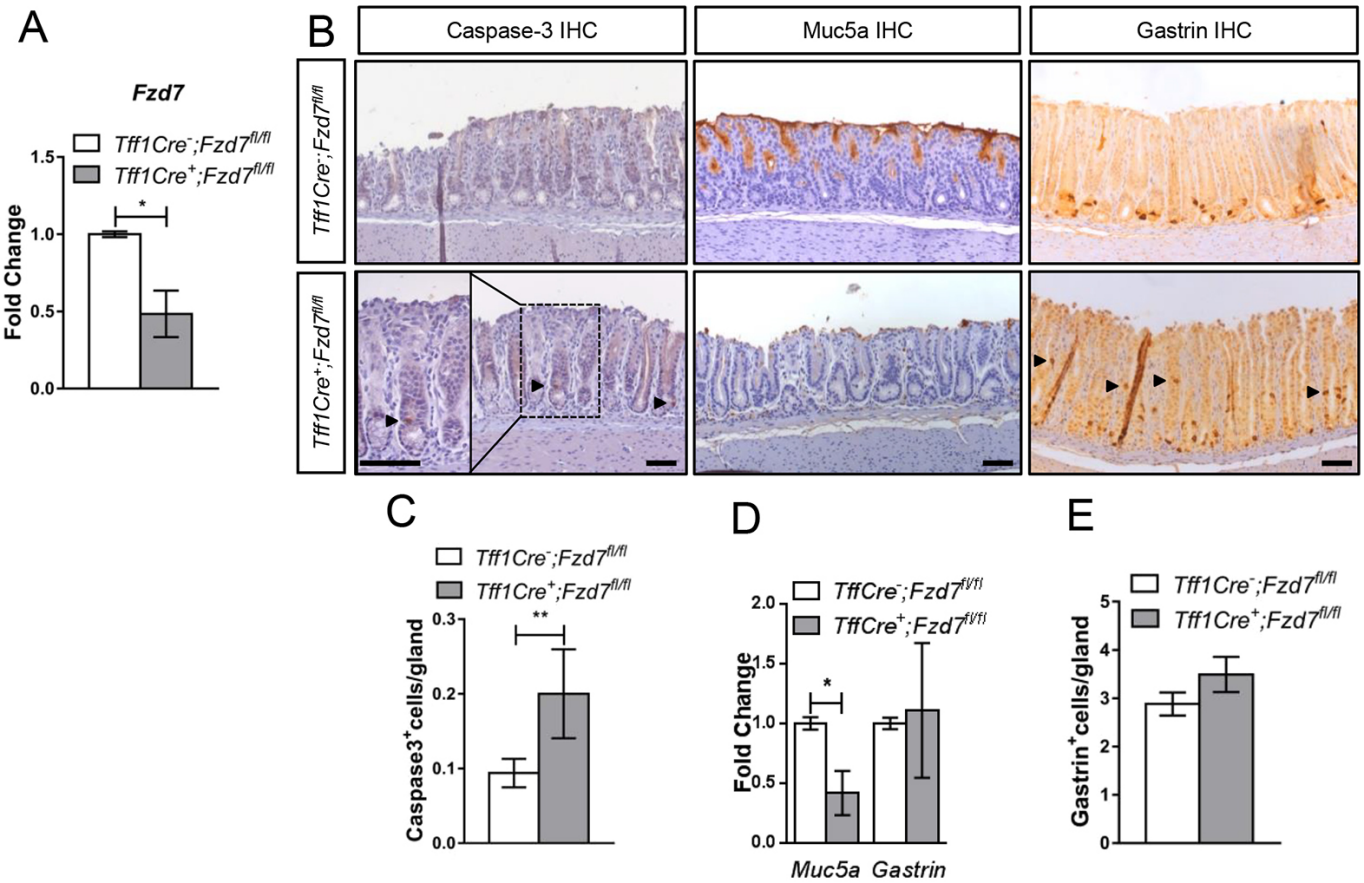
**Deletion of *Fzd7* results in aberrant homeostasis of the antral gastric epithelium.** (A) qRT-PCR on cDNA isolated from the antral epithelium of genotypes indicated 3 days after tamoxifen treatment (*P<0.05; data are mean±s.e.m., *n*=4 mice, Mann–Whitney). (B) Immunohistochemical staining for caspase 3 (indicating apoptosis), Muc5a (indicating mucous-secreting cells) and gastrin (indicating endocrine G cells) on sections from the antral stomach of the genotypes indicated 3 days following tamoxifen injection. The region outlined is shown at higher magnification in the inset. Black arrowheads identify positively stained cells. Scale bars: 100 µm. (C) Enumeration of caspase 3-positive cells from mice indicated in A. Minimum of 40 glands were scored per mouse (*P<0.05; data are mean±s.e.m., *n*=4 mice, Mann–Whitney). (D) qRT-PCR for genes indicated on cDNA isolated from mice of the genotypes indicated 3 days after tamoxifen injection (*P<0.05; data are mean±s.e.m., *n*=4 mice, Mann–Whitney). (E) Enumeration of gastrin-positive cells from mice indicated in A. A minimum of 40 glands were scored per mouse (*P<0.05; data are mean±s.e.m., *n*=4 mice, Mann–Whitney).

Three days after deletion of *Fzd7* in the gastric epithelium, we observe downregulation of *Fzd7* expression 2 days before repopulation is apparent at day 5 (Figs 5A and 4A). We therefore analysed the gastric epithelium to determine the phenotype of deleting *Fzd7* in this tissue. Caspase 3 immunohistochemistry revealed a marked increase in the number of apoptotic cells after *Fzd7* deletion (Fig. 5B,C), which is consistent with our observations in gastric organoid cultures, demonstrating that deletion of *Fzd7* triggers apoptosis in gastric epithelial cells.

Immunohistochemical (IHC) staining for Muc5a revealed that deletion of *Fzd7* resulted in the dramatic reduction of mucus-secreting cells (Fig. 5B), which was associated with a significant reduction in the expression of Wnt target gene *Muc5a* (Mucenski et al., 2005) (Fig. 5D), suggesting that Fzd7 regulates *Muc5a* expression and thus the differentiation of mucus-secreting cells. Immunohistochemistry for gastrin showed that G cells were mislocalised along the length of antral glands following *Fzd7* deletion compared with their usual position towards the base of antral glands in control mice (Fig. 5B). Scoring the number of gastrin-positive cells revealed no significant difference in their numbers in each antral gland, which is consistent with the unchanged expression levels of gastrin transcript between *Fzd7*-deleted and control mice (Fig. 5D,E). To further investigate whether deletion of *Fzd7* was altering the differentiation and function of G cells, we performed immunohistochemistry to visualise expression of the gastric hormones ghrelin and somatostatin. The expression of both hormones was consistent between *Fzd7-*deleted and *Fzd7*-proficient mice in the antrum, strongly suggesting that the mislocalised G cells are still functional (Fig. S6). These data suggest that Fzd7 regulates differentiation of mucus-secreting cells and also the localisation of G cells along the gastric gland.

### Gastric repopulation is characterised by a transient increase in Wnt signalling

**Fig. 6.**
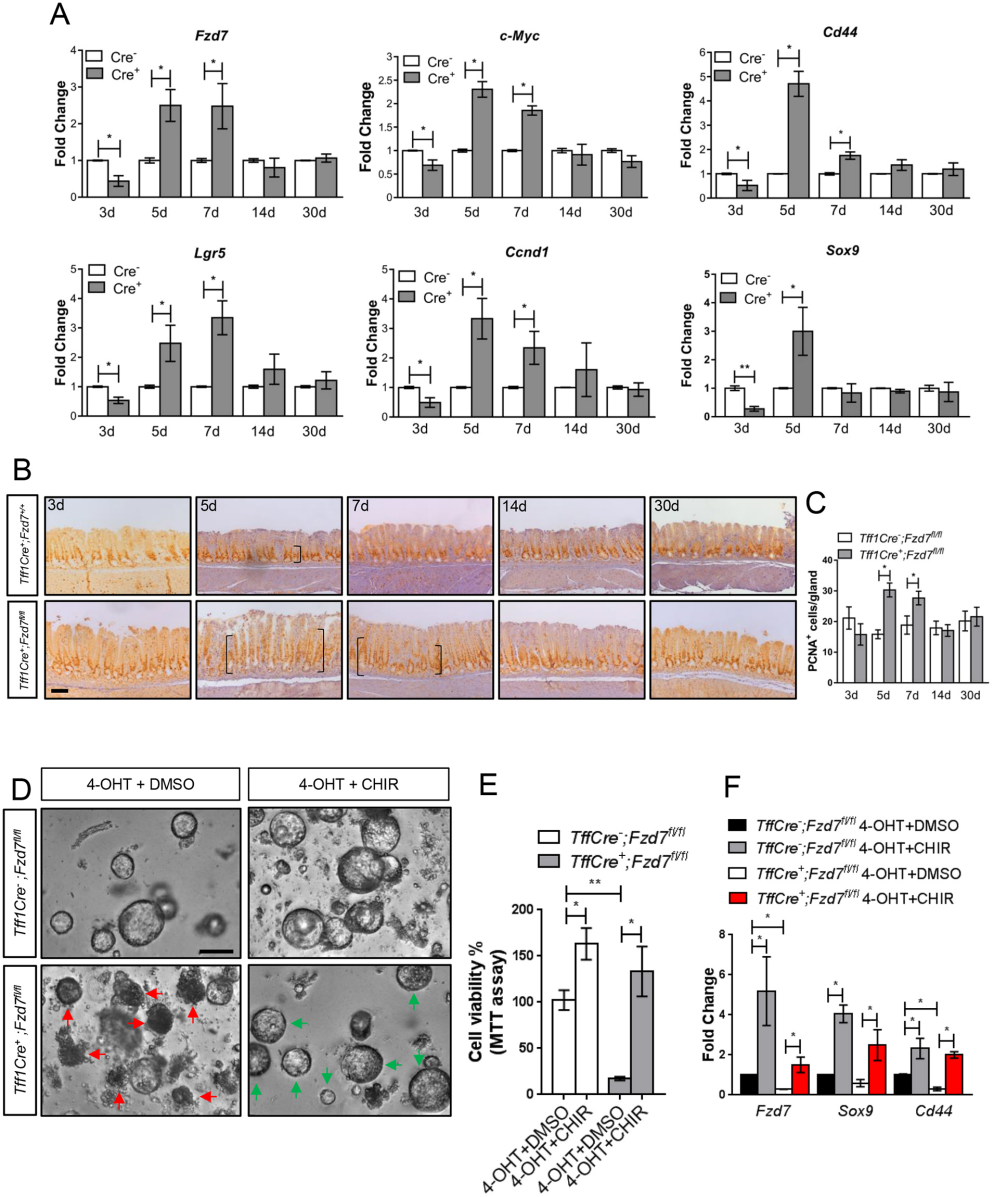
**Wnt signalling regulates gastric repopulation.** (A) qRT-PCR of Wnt/β-catenin target genes from the genotypes and time points indicated (dpi, days post induction) (*P<0.05, **P<0.01; data are mean±s.e.m., *n*=4 mice, Mann–Whitney). (B) Immunohistochemical staining for PCNA (proliferation) on sections from the antral stomach of the genotypes and time points indicated. Brackets indicate stained proliferative zone. Scale bar: 100 µm. (C) Enumeration of immunohistochemistry in 6B (*P<0.05; data are mean±s.e.m., *n*=4 mice, Mann–Whitney). (D) Organoids cultured from the antral epithelium of genotypes indicated and treated with both 4-OHT and vehicle or 4-OHT and CHIR. Green arrows indicate live organoids; red arrows identify dead/dying organoids. Scale bar: 100 µm. (E) MTT assay of cell viability of the organoids described in D. Three mice were used per experimental condition; each experiment was performed separately three times using six replicates of each condition (*P<0.05, **P<0.01; mean±s.e.m., *n*=3 mice, Mann–Whitney). (F) qRT-PCR for Wnt/β-catenin target genes on cDNA isolated from organoids described in D (*P<0.05; data are mean±s.e.m., *n*=3 mice, Mann–Whitney).

To monitor the activation of the Wnt pathway during the gastric repopulation event triggered by *Fzd7* deletion, we examined the expression of several Wnt target genes in the antrum at different time points. All Wnt target genes examined were downregulated 3 days after *Fzd7* deletion, concordant with a reduction in *Fzd7* expression (Fig. 6A) and suggesting that Fzd7 is required to transmit Wnt signalling in this tissue. Surprisingly, 5 days after *Fzd7* deletion, the expression of *Fzd7* and the other Wnt target genes is significantly upregulated, and expression continues to remain high until repopulation has resolved at 14 days (Fig. 6A).

Wnt signalling has been demonstrated to regulate cell proliferation in many tissues and we therefore performed immunohistochemistry for the cell proliferation marker PCNA. Scoring for PCNA-positive cells per gland identified a transient increase in proliferation 5 days and 7 days after *Fzd7* deletion, and a return to wild-type levels of proliferation as repopulation was resolved (Fig. 6B,C). This is consistent with the general gene expression profiles of the Wnt target genes analysed, which shows that Wnt signalling is elevated during this period of repopulation and is restored to wild-type levels by 30 days post *Fzd7* deletion.

These data strongly suggest that Wnt signalling, via *Fzd7*, plays an important role during the process of gastric repopulation following *Fzd7* deletion. To investigate the functional requirement for Wnt signalling downstream of *Fzd7*, we deleted *Fzd7* from gastric organoids and treated them with the Wnt agonist CHIR. The atrophy and cell death observed when *Fzd7* was deleted in gastric organoids was completely rescued when these organoids were additionally treated with CHIR (Fig. 6D). A MTT assay confirmed that the loss of cell viability observed when *Fzd7* was deleted was completely rescued if we then activated the Wnt pathway downstream of the receptor using CHIR treatment (Fig. 6E). Analysis of Wnt target genes *Fzd7*, *Sox9* and *Cd44* by qRT-PCR showed that they were dramatically upregulated in response to CHIR treatment (Fig. 6F). Conversely, these genes were downregulated when *Fzd7* was deleted. However, in *Fzd7*-deleted organoids treated with CHIR, which do not display the atrophy of *Fzd7* deletion alone, the level of these Wnt target genes remained not significantly different from those of the untreated organoids, demonstrating that loss of Wnt signalling is the mechanism responsible for the atrophy and organoid death when *Fzd7* is deleted.

## DISCUSSION

Here, we show for the first time a functional requirement for Wnt signalling in the gastric epithelium via the Wnt receptor Fzd7, and that deletion of *Fzd7* specifically in the gastric epithelium can trigger repopulation of this tissue, which until now has not been reported. Embryonic development of the corpus (fundus in humans) requires active Wnt signalling, whereas inhibition of Wnt results in antral development (McCracken et al., 2017). However, in the adult gastric epithelium, Wnt signalling is active in different areas. The *Axin2-LacZ* mouse shows that Wnt signalling is highest in the base and isthmus of antral glands (Barker et al., 2010; Stange et al., 2013), which is consistent with our data here where we also observe expression of *Fzd7* in this area of the antrum. In addition, Wnt3a is required in the culture medium for gastric organoids, suggesting an important role for Wnt receptors during gastric homeostasis. Although genetic aberrant activation of Wnt signalling can lead to tumourigenesis (Radulescu et al., 2013), there have been no functional experiments to inhibit the Wnt pathway and examine the consequences to gastric homeostasis. To investigate the requirement for Wnt signalling in gastric epithelial cells, we first treated gastric organoids with Wnt inhibitors, IWP-2 or XAV939, which resulted in reduced organoid cell viability. Interestingly, we have previously shown that removal of Wnt3a from the culture media results in organoid death (Barker et al., 2010). However, as IWP-2 is a porcupine inhibitor and thus prevents cells from secreting all Wnt ligands, this strongly suggests that gastric epithelial cells require cell-autonomously secreted Wnt in addition to supplemental Wnt3a provided by the culture media. Thus, our new data implicate a role for both epithelial and underlying stromal cells as a source of Wnt ligands regulating gastric homeostasis *in vivo*. Indeed, deletion of *Fzd7*, which is expressed in the gastric antrum, resulted in cell atrophy and organoid death, similar to organoids treated with Wnt pathway inhibitors. *Fzd7* deletion also resulted in the death of intestinal organoids (Flanagan et al., 2015), suggesting a common role for this receptor in both of these tissues to regulate homeostasis. Interestingly, treatment of gastric organoids with IWP-2 or XAV939 resulted in earlier death of organoids than deletion of *Fzd7*. This could be due to the significant upregulation of *Fzd3* following deletion of *Fzd7*, which is then able to partially and transiently compensate for the loss of *Fzd7*, which would be ineffective in organoids treated with IWP-2 or XAV939 as they block the Wnt pathway at the level of Wnt secretion or β-catenin, respectively (Fig. S1C). This is similar to the situation we previously reported in the small intestine in which deletion of *Fzd7* is partially compensated for by upregulation of *Fzd1* and *Fzd2* during intestinal regeneration (Flanagan et al., 2015).

Surprisingly, when we deleted *Fzd7* from the gastric epithelium *in vivo*, we did not observe widespread atrophy and denuding of the epithelium, as might be expected. However, using lineage tracing of recombined *Fzd7*-deficient cells, we were able to track a repopulation event in the gastric epithelium for the first time, in which non-recombined, *Fzd7*-proficient cells replaced the *Fzd7*-deficient cells over the course of 7-10 days. This is consistent with previous lineage tracing showing full glands could be generated from *Lgr5*^+^ cells in the same way in 7-10 days (Barker et al., 2010). Repopulation does not occur from a denuded epithelium, as in regeneration, and therefore does not preclude that a large apoptotic event is associated with it. We and others have previously reported that the intestinal epithelium is able to repopulate after deletion of crucial genes such as *Myc* (Muncan et al., 2006), *Stat3* (Matthews et al., 2011) or *Chek1* (Greenow et al., 2009). Indeed, we also recently observed repopulation when we deleted *Fzd7* in the intestinal epithelium, again suggesting a common role for this receptor in gastric and intestinal homeostasis. This mechanism of repopulation is an important adaptation to allow these epithelial layers to rapidly respond to damaging molecular events that could otherwise disrupt the delicate homeostasis of these tissues, resulting in possible pathologies that include colitis/gastritis and neoplasia (Clevers et al., 2014). Repopulation of the gastric epithelium with bone marrow-derived cells (BMDCs) has been previously reported *in vivo*, but only 30 weeks after experimental infection with *Helicobacter*, which eventually resulted in the development of gastric tumours, with no repopulating cells observed at earlier time points (Houghton et al., 2004). This then represents a very different kind of repopulation to the rapid event we describe here, which results in the gastric epithelium returning to a normal homeostatic state after the repopulation event, rather than any associated pathology as observed with the slow BMDC repopulation. Epithelial damage and gastric ulceration are common pathologies associated with radiotherapy in humans (Coia et al., 1995; Henriksson et al., 1999). This suggests that manipulation of the Wnt pathway following irradiation could be of therapeutic benefit for individuals receiving radiotherapy, as has been suggested in the intestine (Ashton et al., 2010; Phesse and Sansom, 2013; Zhou et al., 2013).

Gastric repopulation is not observed until 5 days after *Fzd7* deletion. Therefore, before this time point we can analyse the requirement for Fzd7 in the gastric epithelium. At 3 days post-deletion we could demonstrate robust deletion of *Fzd7* from the gastric epithelium, observed as perturbed differentiation of Muc5a^+^ mucus-secreting cells, which was also previously reported in mice with hyperactive Notch signalling (Demitrack et al., 2015). However, neither inhibition nor hyperactivation of Notch signalling triggered repopulation in the gastric epithelium, suggesting that this mechanism is exquisitely sensitive to loss of Wnt signalling. These data suggest that Notch and Wnt signalling work in parallel to regulate gastric homeostasis, with distinct functions from one another. In support of this, proliferation is also altered in the gastric epithelium in response to modulated Notch signalling (Demitrack et al., 2015; Kim and Shivdasani, 2011), which we did not observe in the *Fzd7*-deficient gastric epithelium 3 days after *Fzd7* deletion. Transient changes in proliferation were observed only during the gastric epithelial repopulation event, which was characterised by the return of *Fzd7*-proficient cells and increased Wnt activation. A small increase in the number of apoptotic cells per gland was also observed following *Fzd7* deletion. These apoptotic events were located in the isthmus of antral glands, which is the location of a population of stem cells marked by either *Lrig1*, *Sox2* or *Cckbr* (Hayakawa et al., 2016). This suggests that deletion of *Fzd7* may be deleterious to these stem cells, and consequently triggers repopulation, which will be important to investigate in future studies. Furthermore, *Lgr5*^+^ cells are located in the base rather than in the isthmus of the antrum, suggesting that, in contrast to the intestine, Fzd7 may be regulating a population of stem cells that are not expressing *Lgr5*.

Regulation of differentiation by Wnt signalling is also observed in the intestinal epithelium, where activation or inhibition of the pathway can result in perturbed differentiation and mislocalisation of Paneth cells (Phesse et al., 2008; Sansom et al., 2004). Indeed, G cells are mislocalised throughout the gastric antral glands after *Fzd7* deletion, rather than located at their usual position at the base of the glands. These data strongly suggest a conserved function for Wnt signalling in regulating the location of differentiated cells within the gastric and intestinal epithelium.

Deletion of *Fzd7* perturbs gastric organoid viability. In contrast, treatment of gastric organoids with the Wnt pathway activator CHIR, which inhibits Gsk3β, increases Wnt target gene expression and cell viability. These data identify Wnt as an important regulator of gastric epithelial cell function. Intriguingly, the deletion of *Fzd7* in these gastric organoids prevents CHIR treatment from activating Wnt target genes to the levels observed in *Fzd7*-proficient organoids. These results demonstrate that modulation of Wnt/Fzd receptor interactions can still influence the outcome of cells in which the cytoplasmic downstream signal transducers of the pathway have been mutated. This is consistent with previous findings in which we and others have shown that Wnt pathway activity can still be modulated in colon cancer cells with mutant APC (Caldwell et al., 2004; Suzuki et al., 2004; Vincan et al., 2007, 2005). As the Wnt pathway is also deregulated in gastric cancer (Phesse et al., 2016), these data also suggest that Fzd receptors could be a target for therapeutic intervention for this disease.

## MATERIALS AND METHODS

### Mice

The BAC transgenic Tg(*Tff1Cre^ERT2^*) (Thiem et al., 2016), *Fzd7^fl/fl^* (Flanagan et al., 2015), *Fzd7^nLacZ^* (Yu et al., 2012), *Fzd5^fl/fl^* (van Es et al., 2005) and *Rosa26LacZ^LSL^* (Soriano, 1999) have all been previously described. Mice were interbred to generate compound mice with appropriate alleles. All mice were co-housed, and with the exception of *Fzd7^nLacZ^* mice, all mice were on an inbred C57Bl/6 genetic background, using males and females and appropriate littermates as controls. The *Fzd7^nLacZ^* mice were on a mixed C57Bl/6×Sv129 background. All animal experiments were approved by the Animal Ethics Committee, Office for Research Ethics and Integrity, University of Melbourne, Australia.

### Tamoxifen administration

Short-term *in vivo* Cre induction (<7 days post induction) was performed in 6- to 10-week-old mice with a single intraperitoneal (i.p.) injection of 2 mg of tamoxifen per mouse. Long-term *in vivo* Cre induction (>14 days post induction) was performed in 6- to 10-week-old mice with a single daily i.p. injection of 2 mg of tamoxifen per mouse per day over four consecutive days.

### β-Galactosidase (X-gal) staining

Freshly isolated stomachs were cut along their greater curvature, washed with PBS and immediately fixed (1% formaldehyde, 0.2% gluteraldehyde, 0.02% NP-40 in PBS) for 2 h at 4°C. The fixative was removed and stomachs were washed in PBS. Stomachs were incubated in β-galactosidase detection substrate [5 mM K_3_Fe(CN)_6_, 5 mM K_4_Fe(CN)_6_.3H_2_0, 2 mM MgCl_2_, 0.02% NP-40, 0.1% sodium deoxycholate, 1 mg/ml X-gal in PBS] in the dark, overnight at room temperature. The detection substrate was removed the following day and stomachs were washed in PBS, followed by an overnight incubation in 4% PFA at 4°C in the dark. The PFA was removed and stomachs were washed in PBS. Stained stomachs were placed into histological cassettes, embedded in paraffin wax, sectioned at 5 µm, mounted onto slides and counterstained with Neutral Red.

### Tissue collection and histological analysis

Freshly isolated mouse stomachs were flushed with PBS and fixed overnight at 4°C in 10% neutral buffered formalin (NBF) and washed twice in 70% ethanol at room temperature. Tissues were placed into histological cassettes, embedded in paraffin wax, sectioned at 5 µm and mounted onto slides as described previously (Flanagan et al., 2015). Paraffin sections were de-waxed, re-hydrated, blocked and incubated in primary antibody overnight at 4°C. Sections were washed and incubated in secondary antibody (polymer horse radish peroxidase-conjugated mouse/rabbit/goat) for 30 min at room temperature. Sections were rinsed in and bound peroxidase was detected and developed by adding diaminobuteric acid substrate (DAB) at room temperature. Slides were washed in MilliQ water and nuclei counterstained with Mayer’s haematoxylin. Antibodies used were mouse anti-Muc5aC (1:400, Thermoscientific, MS-145B0), rabbit anti-PCNA (1:300, Santa Cruz, SC-7907), rabbit anti-caspase 3 (1:1000, R&D systems, AF-835) and goat anti-gastrin-C20 (1:400, Santa Cruz, SC-7783).

### Isolation and culture of gastric organoids

The stomachs from mice were dissected out, cut along the greater curvature and flushed in ice-cold PBS, then incubated in a 50 mM EDTA (pH 8.0) in PBS chelating solution for 1 h on a roller at 4°C. Stomachs were then transferred to tubes containing PBS and vigorously shaken to dissociate gastric glands from the underlying stroma (Flanagan et al., 2016). Isolated gastric gland suspension was filtered through a 70 µM cell strainer (BD Biosciences, #352350), which was collected and counted using a haemocytometer. The gastric glands were resuspended in Matrigel (∼100 glands/50 µl of Matrigel) and plated onto a 24-well tissue culture plate. Once the Matrigel had set at 37°C, organoids were covered with 500 µl of gastric culture medium as previously described (Barker et al., 2010; Flanagan et al., 2016). Gastric medium containing growth factors was replenished every other day and cultures were passaged and split once a week. *In vitro* Cre recombinase was activated by treating gastric organoid cultures with 100 nM 4-hydroxytamoxifen (4-OHT) as previously described (Barker et al., 2010; Flanagan et al., 2016). Organoid cultures were imaged on a Nikon Ti-E microscope using DIC (differential interference contrast) with a 4× PlanApo NA 0.3 objective. A focal stack of images was collected 10 µm apart and processed through the ‘Best Focus’ function of MetaMorph v7.7.7 (Molecular Devices) to generate the final image of individual organoids as previously described (Flanagan et al., 2015; Phesse et al., 2014).

### RNA extraction and analysis

Gastric glands were homogenised in TRizol and total RNA was purified and DNAse treated on Qiagen columns (Promega) and quantified using a DNA/RNA nanodrop spectrophotometer. Four μg of each RNA sample was reverse transcribed using anchored oligodT primers (Promega) and Moloney Murine Leukemia Virus Reverse Transcriptase (M-MLV RT, Promega, #M1705) following the manufacturers' instructions in a final volume of 100 µl, as previously described (Vincan et al., 2007). Real-time RT–PCR was performed using the SYBR green PCRmaster mix and the ABI PRISM 7500 sequence detection system (Applied Biosystems) on cDNA synthesized from DNase-treated total RNA as previously described (Flanagan et al., 2015). Gene expression levels were calculated relative to the housekeeping gene 18S. The 2-^ΔΔCT^ method (Bustin et al., 2009) was used to calculate the fold change as previously described (Phesse et al., 2008; Vincan et al., 2007). Primer sequences are available in Table S1.

### MTT assay

Following treatment, gastric organoids were mechanically dissociated, washed with ADF, counted, resuspended in fresh Matrigel and seeded in a flat-bottomed 96-well tissue culture plate and incubated for 24 h at 37°C in a 5% CO_2_ chamber. Organoids were incubated with MTT (thiazolyl blue tetrazolium bromide, Sigma, #M2128) for 4 h at 37°C in 5% CO_2_ chamber. Gastric culture medium was removed from organoids and replaced with lysis buffer (50% DMF, SDS, acetic acid+2.5% 1 M HCl) and incubated overnight at 37°C. Solution (100 µl) was transferred to a clean flat-bottomed 96-well plate and optical density determined using BMG lumistar plate reader (Hansen et al., 1989).

### Statistical analysis

Data are expressed as mean±s.e.m., where mean represents number of mice (≥3 per genotype) or number of independent experiments (≥3). Statistical tests used were Mann–Whitney with Prism7 (GraphPad software) where *P*≤0.05 was considered significant.
